# Laser Modification of Functional Fibers Obtained by Electrospinning

**DOI:** 10.3390/ma18245631

**Published:** 2025-12-15

**Authors:** Anna Firych-Nowacka, Mariusz Tomczyk, Ewa Korzeniewska, Magdalena Grala

**Affiliations:** 1 Institute of Mechatronics and Information Systems, Lodz University of Technology, 22 Stefanowskiego Street, 90-537 Łódź, Poland; anna.firych-nowacka@p.lodz.pl; 2 Institute of Electrical Engineering Systems, Lodz University of Technology, 18 Stefanowskiego, 90-537 Łódź, Poland; mariusz.tomczyk@p.lodz.pl; 3 Department of Chemical and Molecular Engineering, Lodz University of Technology, Wólczańska 213 Street, 93-005 Łódź, Poland; magdalena.grala@p.lodz.pl

**Keywords:** functional fibers, micro- and nanofibers, electrospinning, laser treatment, laser beam

## Abstract

In this article, the authors present the impact of laser treatment on the structure of magnetic composite microfibers. Changes occurring on the surface can have a significant impact on the conductive properties of functional materials produced on a micro- and nanoscale. The fibers presented are functional materials that gain technical applications when combined with other materials. In this case, we refer to the concept of textronics, i.e., the combination of textiles with electronics to create various types of flexible sensors. The authors performed microscopic analysis to observe the changes occurring in the materials. For this purpose, scanning electron microscope and atomic force microscope were used.

## 1. Introduction

In recent years, electrospinning has garnered considerable interest as a method for producing fibers with nanometer diameters, which are utilized in various fields, including medicine, materials science, and the textile industry [1]. This technological process is used to produce functional fibers, meaning those exhibiting properties beyond purely mechanical ones, such as antibacterial, hydrophilic, conductive, or biocompatible properties.

Typical electrospinning is a process in which a base polymer solution is transformed into thin fibers by the action of an electric field [2]. In this technological process, a drop of solution contained in a syringe forms a Taylor cone at the tip of the needle under the influence of an electric field [3]. When electrostatic forces overcome the surface tension, the solution is ejected towards a metal plate or rotating cylinder acting as a collector, creating fibers with microscopic diameters [4]. According to Suresh et al., the mutual arrangement of the parts of the device used in the electrospinning process and gravity have a significant impact on the entire process, directly influencing the shape of the Taylor cone, the trajectory of the flowing polymer stream, as well as the diameter of the produced fibers, their distribution, and the overall spinning efficiency [5]

The resulting fibers can be modified at any stage of the production process (Table 1). Modification of the polymer solution by adding functional nanoparticles such as Ag, ZnO, and TiO_2_ imparts antibacterial, antiviral, and photoactive properties to the fibers at the structural level [6,7], while the addition of Fe_3_O_4_ and CNTs (carbon nanotubes) enables the achievement of electrically conductive or magnetic properties. Adding natural polymers, such as chitosan or gelatine, to the solution ensures the biocompatibility and biodegradability of these fibers [8,9,10], while adding dyes or pigments imparts optical properties to the fibers [11]. Adding components to the base polymer at the initial stage of the electrospinning process enables the uniform distribution of functional particles throughout the fiber cross-section.

Functional fibers created by electrospinning can be modified at various stages of their production. The goal of such modifications is to adapt their structure, surface properties, and functionality to the desired electrical and magnetic properties. Modifications are achieved by adjusting the electrospinning process parameters, adding additives during fiber production, and using post-modification techniques to achieve the desired properties [3,15].

Utilizing the physical and structural capabilities of the electrospinning process during its course enables the creation of complex structures and control of the substance release profile. Controlling process parameters such as voltage, needle-collector distance, polymer flow rate, and temperature influences the thickness, porosity, and creation of specialized structures using the produced fibers [8,9,12,13,14]. The core-shell fibers, where the active substances are protected by the creation of an additional outer layer, can be produced by modifying process parameters.

After forming the fibers, they can be subjected to a range of treatments that impart additional functionality. This is achieved by subjecting the fibers to plasma treatment, which modifies the functional groups on their surface, resulting in, for example, improved hydrophilicity or biological binding capacity [16,17]. Another well-known process that modifies fiber properties is curing them with UV radiation, heat treatment or chemical cross-linking agents (e.g., glutaraldehyde), which increases the mechanical resistance of electrospinning products or changes their morphology [12,13]. Micro- and nanofibers produced by electrospinning can also be impregnated or coated with active layers, thus achieving surface biological or detection activity, enabling the application of antibiotics, antibodies, enzymes or the use of the fibers as environmental sensors [18]. Perez et al. also found that lasers with wavelength output at 1065 nm effectively cleaned wood pulp paper, which consists of cellulose, and removed dirt layers, reducing filler migration and color change [19].

Each modification of fibers obtained through electrospinning opens the door to various applications. In medicine, they can be used in dressing materials, tissue scaffolds, drug delivery systems or pathogen, antibiotic detection, and pesticide residue detection [20,21,22,23,24]. In electronics, functional nano- and microfibers are used as sensory elements or electrically conductive layers, while in functional textiles, they are used in protective clothing or thermoregulating materials [8,12,13,25].

Previous studies have utilized laser beam interaction to modify electrospun fibers during the process. The production of thinner fibers increased molecular orientation, and higher crystallinity is allowed using a laser to locally heat the polymer during electrospinning (laser-assisted melt electrospinning) [26]. Fibers with diameters as small as 500 nm and tailored structural properties can be created by adjusting the laser power and spinning parameters [27,28]. According to Tokuda et al., laser-assisted electrospinning allows for the production of ultrathin fibers with an average diameter of 1 μm, potentially useful in various applications, such as packaging materials and battery separators [29]. Higher laser power used in this process can result in a greater number of generated polymer jets, further reducing the fiber diameter and increasing production efficiency [29]. According to Lupu et al., it is possible to obtain fibers that are 20 percent thinner and have a higher density using laser diodes in the electrospinning process [30].

Cheng et al. subjected laser-sensitive polycarbonate (PC) fibers obtained by electrospinning to a porous structure by scanning with a pulsed beam in air. During the laser irradiation process, they observed a series of changes, including melting, thermal degradation, and carbonization of the polymer fibers, resulting in variable surface structures. The results presented by the group indicate that porous carbon structures can be created on the surface of the fibrous membrane by controlling the degree of laser-induced carbonization, which translates into a high adsorption efficiency of unwanted xylene [31].

Seeking other possibilities for modifying fibers produced through the electrospinning process, the authors of this paper researched the effect of laser beam exposure on the structure of micro- and nanofibers produced by the classic electrospinning method. The authors considered the modification of the surface structure of the produced fibers in terms of the possibility of obtaining the desired roughness.

## 2. Materials and Methods

### 2.1. Materials and Electrospinning Process

Composite cellulose fibers subjected to laser treatment, as described in this article, are materials consisting of cellulose—the main component of plant cell walls—combined with other substances or structures, forming fibers with improved mechanical, thermal, or functional properties compared to cellulose alone. These magnetic microfibers are a composite in which a polymer with a powder filler forms a discontinuous phase. They are produced from cellulose into which magnetic powder is incorporated.

The technique employed to obtain fibers exhibiting magnetic properties was the Lyocell process. This method involves the production of fibers from concentrated solutions of cellulose (Cellulose DP ≈ 500, Sigma-Aldrich, Poznań, Poland) in N-methylmorpholine N-oxide (NMMO, Sigma-Aldrich, Poznań, Poland)—characterized by its molecular formula C_5_H_11_NO_2_ and a molecular weight of 117.15 g/mol. To achieve the desired magnetic response, modifiers possessing hard and soft magnetic characteristics are incorporated into the spinning solutions. Modification conducted at the stage of spinning solution preparation represents one of the most effective approaches to imparting new functionalities to fibers, as it enables a homogeneous distribution of modifier particles throughout the entire fiber volume. The incorporation of these particles into the cellulose matrix ensures a stable and durable modification effect, in contrast to techniques based on surface treatment [32].

In the case of the microfibers presented in this study, the Lyocell process commenced with the dissolution of cellulose in NMMO, resulting in the formation of a viscous solution. A 60% aqueous solution of NMMO was employed (prepared from NMMO·H_2_O monohydrate), which was capable of directly dissolving pure cellulose without any chemical or structural modification. The resulting spinning solution was then prepared for fiber formation using the electrospinning process. After collection on the rotating drum, NMMO was leached from the fibers in water, and the cellulose re-solidified.

At the final stage of the process, the NMMO solvent was recovered and recycled, with an efficiency exceeding 99%. Consequently, the Lyocell method is regarded as an environmentally sustainable and eco-friendly technology. The monohydrate form (NMMO·H_2_O) was used, as it provides the right balance of solvency and thermal stability for dissolving cellulose.

Cellulose fibers were produced using a combination of hard and soft magnetic materials, and the resulting samples were analyzed in the study. Barium ferrite (BaFe_12_O_19_), synthesized from 96% purity iron oxide Fe_2_O_3_ (Warchem Sp. z o.o., Zakręt, Poland) and 98% purity barium carbonate—BaCO_3_ (Warchem Sp. z o.o., Zakręt, Poland)—was chosen as the hard magnetic component. This powder was dispersed in the NMMO solvent using a high-shear mixer (IKA T25 digital, IKA, Staufen, Germany) operating at 10,000 rpm for 40 min and then subjected to ultrasound (Bandelin Sonorex RK 100, Bandelin, Berlin, Germany) for 45 min to ensure homogeneity before electrospinning the formation of fabrics. This is crucial for breaking up agglomerates and obtaining a stable, homogeneous suspension ready for the process.

The soft magnetic component consisted of a nanocrystalline alloy derived from the controlled crystallization of metallic glasses with the composition Fe-M B (where M includes elements such as Nb, Cu, Hf, Zr, and Si). Both types of magnetic modifiers were added to the spinning solution in powdered form, with an average particle size of 8 μm. This modification process enabled a significant incorporation of magnetic materials into the fiber matrix—up to 50% by weight for the hard magnetic filler and up to 40% for the soft magnetic one. These values represented the maximum filler content that could be successfully processed using standard spinning techniques. Two microfiber samples that were analyzed in the paper were prepared containing 10% (sample no. 1) and 23% (sample no. 2) hard magnetic material, respectively. The fiber formation was carried out using the electrospinning process at a take-up speed of 70 m/min.

A cellulose concentration of 8% (*w*/*w*) in NMMO was used for the electrospinning process. The resulting solution exhibited a viscosity of 1800 mPa·s at 80 °C, a key parameter for stable jet formation during electrospinning. The applied voltage was 15 kV, and the distance between the needle and the collector was 15 cm.

**Figure 1:**
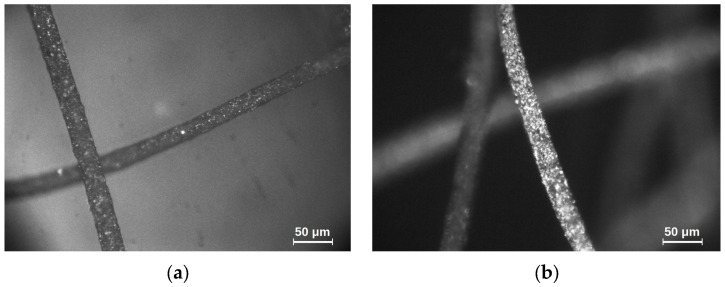
Magnetic microfiber images taken with an optical microscope: (**a**) sample no. 1—ferromagnetic volume filling 10%, and (**b**) sample no. 2—ferromagnetic volume filling 23%.

The tests conducted concern magnetic microfibers, in which the ferromagnetic volume filling was 10% in the first case and 23% in the second, as shown in Figure 1 below. The images were taken with an optical microscope at 200× magnification using the reflected light technique.

### 2.2. Method of Laser Treatment

Cellulose is a biopolymer with a fibrous structure characterized by specific physical and chemical properties dependent on its structure. Due to its optical properties, pure cellulose does not absorb near-infrared (NIR) laser radiation, including that emitted by the 1065 nm fiber laser used. In the described research, cellulose micro- and nanofibers doped with barium ferrite (BaFe_12_O_19_) were laser-processed. The absorption properties of this composite for a given laser wavelength are determined by the percentage of iron content. The absorption coefficient for 1065 nm laser radiation for iron is approximately 0.4 (the remaining radiation is reflected). This coefficient depends on the temperature, oxidation state, fiber surface roughness, and laser beam parameters. The level of absorbed energy is sufficient to initiate physicochemical transformations in the composite.

For laser processing of cellulose fiber doped with barium ferrite, a fiber laser emitting radiation (SPIG3, SPI Lasers Ltd., Southampton, UK) at a wavelength of 1065 nm was selected. A pulsed beam with a pulse duration of 15 ns was used. Pulses were repeated at a frequency of 290 kHz, and the beam travelled at a speed of 1000 mm/s. In the cross-section, the surface power density distribution was Gaussian, and the focused beam diameter was 26 mm. For the specified pulse repetition frequency, beam scanning speed, and beam diameter, an overlap of 22 mm was obtained. This means that the laser beam struck the fiber surface eight times in each interaction zone. Multiple exposure of laser pulses to the same location produces an energy concentration effect, improving the efficiency of laser processing. The energy of individual pulses varied within a range from 0.0102 mJ to 0.034 mJ, controlled by laser power (from 3 W to 10 W). Delivering this energy within 15 ns allows for very high surface power densities ranging from 1.28 × 10^12^ to 4.27 × 10^12^ W/m^2^, respectively (Table 2). Such power levels are only possible for laser processing.

The laser beam was scanned transversely to the fiber axis with a hatching of 0.01 to 0.05 mm. Large spacings (0.05 and 0.03 mm) did not ensure complete coverage of the fiber surface with the laser beam.

In the first stage of laser radiation, energy is absorbed by the material’s surface at a depth of a few micrometers. The next stage is heat transport by conduction, described by the well-known Fourier equation. During the pulse, a plasma cloud is created, which increases energy absorption.

Laser irradiation of cellulose-iron nanofibers induces laser pyrolysis, a very rapid, high-temperature process that occurs under controlled conditions. Iron particles in such fibers strongly absorb laser energy and then rapidly transfer this energy to the surrounding cellulose matrix. This leads to a local, rapid temperature increase, much faster than in conventional pyrolysis. The presence of iron can lower the activation energy of thermal cellulose decomposition, accelerating the process. Cellulose decomposes at high temperatures in two ways. The first is dehydration and carbonization, while the second is depolymerization, which leads to the formation of levoglucosene [33,34] and other volatile products such as water, carbon monoxide, and carbon dioxide [35]. The mechanisms of cellulose degradation in the context of conventional heating were described by [35,36]. They provide a background for the processes occurring in the composite, and the iron contained therein accelerates and modifies the thermal decomposition process of cellulose. The created gases escape under high pressure, creating characteristic craters on the fiber’s surface. The optical effect of the laser’s action is the formation of pores on the fiber’s surface and a change in the surface topography.

### 2.3. SEM Analysis Sample Preparation

The morphology of the microfibers was examined using a scanning electron microscope (SEM), Apreo 2S (Thermo Fisher, Waltham, MA, USA), equipped with an energy-dispersive X-ray spectroscopy (EDS) system (EDAX). The samples were placed onto an SEM specimen holder using double-sided carbon tape for electrical conductivity. No sputter coating was applied before SEM analysis. Imaging was conducted in high-vacuum mode at an accelerating voltage of 1 keV, with magnifications of 1000× and 3500×. Both the Everhart-Thornley secondary electron detector (ETD) (Thermo Fisher, Waltham, MA, USA) and the T1 segmented lower in-lens, backscattered electron detector (Thermo Fisher, Waltham, MA, USA) were employed to capture detailed surface features. Two samples were subjected to image analysis. The first sample had a ferromagnetic content of 10% by volume, while the second sample had a ferromagnetic content of approximately 23%.

Elemental composition of selected samples (sample No. 2: 0 W and 3 W) was analyzed by EDS at an accelerating voltage of 3 keV, with spectra acquired from three distinct points on each sample.

### 2.4. AFM Analysis Sample Preparation

The roughness and topography of the samples were investigated by atomic force microscopy. Samples were mounted on a steel specimen disc using double-sided carbon tape to provide proper adhesion and mechanical stability during imaging. No additional chemical treatment or metallization was required. The measurements were obtained under ambient conditions using a MultiMode8-HR atomic force microscope (AFM, Bruker, Billerica, MA, USA). The imaging was performed in tapping mode using a silicon cantilever (RTESPA-300, Bruker, Billerica, MA, USA) with a nominal spring constant of 40 N/m and a nominal tip radius of 8 nm. Areas of 3 µm × 3 µm were scanned at a resolution of 512 × 512 data points and a scan rate of 1 Hz.

### 2.5. Roughness Analysis

The surface roughness was quantitatively analyzed using NanoScope Analysis 3.0 software (Bruker, Billerica, MA, USA). All roughness measurements were carried out in accordance with international standards [37,38].

The surface roughness parameters were evaluated based on the AFM images and included the following:

- R_a_—the arithmetic average height of the profile’s absolute deviations from the mean line,
- R_q_—the square root of the average squared deviations,
- R_max_—maximum vertical distance between the highest and lowest data points (R_p_ + |R_v_|),
- R_v_—the vertical distance from the mean line to the deepest valley,
- R_p_—the vertical distance from the mean line to the highest peak.

The roughness parameters were calculated based on all points measured, i.e., in an area of 3 µm × 3 µm, the microscope measured 512 points in each of the 512 lines. The mean values of the roughness parameters were calculated from 3 randomly selected areas.

## 3. Results

In this section, the authors present changes in the structure of magnetic microfibers, roughness parameters, and iron content spectra before and after laser treatment.

### 3.1. SEM Imaging and Chemical Composition Analysis

The morphology of samples no. 1 and no. 2, treated with different laser powers, can be compared based on the SEM images (Table 3) at 1000× magnification. Table 4 and Table 5 present images for samples no. 1 and no. 2, respectively, taken at 3500× magnification using two types of detectors: ETD and T1. The ETD detector reveals morphological changes, while the T1 segmented lower in-lens detector shows not only morphological changes but also variations in material composition—heavier elements appear distinctly brighter. The better contrast in the T1 detector reveals the grains of ferromagnetic powder implemented into the cellulose fiber.

SEM image analysis revealed that the geometry of cellulose fibers, such as diameter and length of the samples, did not change after laser treatment in the analyzed power range (from 3 W to 5 W). At a power of 3 W, a slight smoothing of the fibrous surface structure of cellulose was noticeable. This effect may indicate depolymerization of cellulose, which can be confirmation of the research achieved by Kolar [39]. According to that research, laser irradiation at this length of laser wave could cause an increase in the degree of polymerization because the formation of inter- and intra-molecular ether bonds can occur. Such effects cannot be observed in microscopic images. Further increases in laser power cause the formation of distinct pores, which are the result of local temperature increases and gas release. A similar mechanism was observed during the iron-catalyzed laser-induced graphitization (IC-LIG) of organic substrates (wood, cellulose, lignin or bark) [40] and during CO_2_ laser treatment of modified cellulose paper treated with a composite solution containing tannic acid, iron(III) citrate, glycerol, and gum Arabic [41]. Craters were formed in areas of strong laser radiation absorption by iron particles. The laser energy also revealed magnetic material crystals hidden beneath the cellulose surface. For processing with a 4 W and 5 W laser beam, the surface roughness increased, as confirmed by further studies presented in Section 3.2. A laser beam power above 5 W caused total destruction of the sample, which was the result of strong ablation and sublimation of the material.

When comparing images of fibers with contents of 10% and 23% (Table 4 and Table 5), the difference in roughness was visible. Samples with a higher iron content had more gas bubbles, regardless of the power used. This confirms the indirect effect of the laser on cellulose through iron grains.

Based on image analysis performed using a SEM microscope, the article presents a qualitative analysis of the spectrum of chemical elements. For this purpose, only sample no. 2 with a ferromagnetism content of 23% by volume was analyzed.

**Figure 2:**
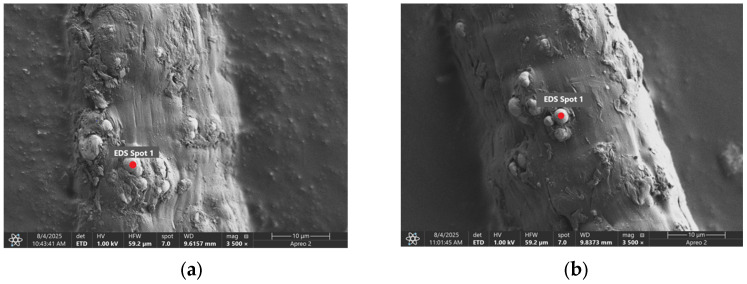
The location of the measuring probe for sample no. 2 with a ferromagnetic content of 23%: (**a**) before laser treatment, and (**b**) after laser treatment (3 W).

The Figure 2 shows the measurement points used to determine the distribution of chemical elements in a magnetic microfiber with a ferromagnetic content of 23%.

**Figure 3:**
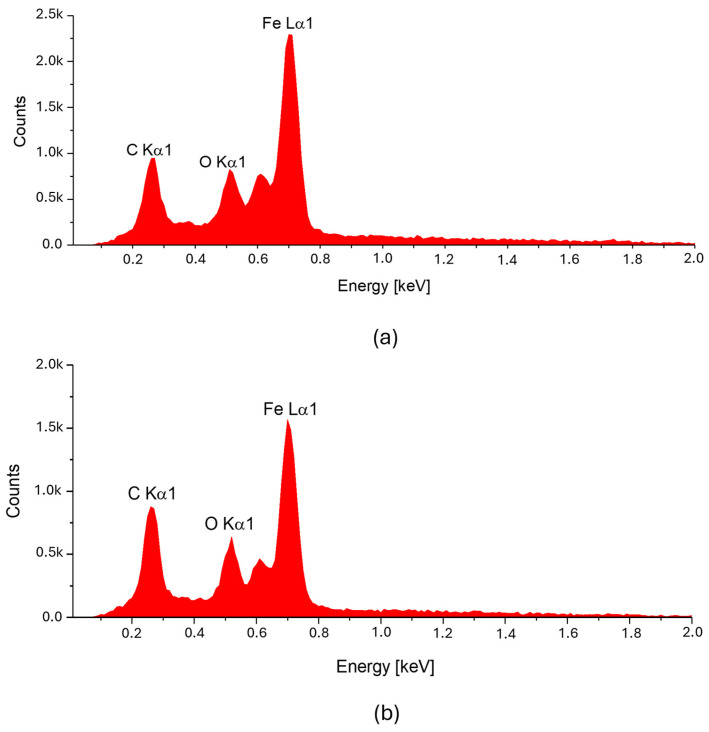
Spectrum of chemical element composition at the selected point of a magnetic microfiber directed at the ferromagnetic grain: (**a**) before laser treatment, and (**b**) after laser treatment (3 W).

The spectrum of chemical elements (Figure 3) at a selected point on the microfiber confirmed the presence of the iron magnetic grains in both samples: before and after laser treatment. Furthermore, no significant changes were observed in the elemental spectra after laser treatment, indicating that the elemental composition remained stable. It can be concluded that the ferromagnet does not degrade, so it is anticipated that such treatment has a limited impact on the electrical and magnetic parameters of such a fiber. As a result of laser treatment, iron grains are exposed on the surface of cellulose.

### 3.2. AFM Imaging Analysis

The examination of the roughness of the fiber structure is very important, as has already been demonstrated in [42]. The authors proved that higher roughness parameters results in a smaller nominal contact area between surfaces, and adhesion to a rough surface is reduced. The analysis of the surface roughness of individual micro- and nanofibers enables the selection of fabrics based on the properties of electrically conductive layers, which are used in further processing in textronics or other electrical applications.

The following figures show samples measured with an atomic force microscope (AFM) at an area of 3 µm × 3 µm (Table 6).

When the laser power was below 3 W, corresponding to an energy of 1.28 × 10^12^ W/m^2^, no material changes were observed in the sample. This means that the fluence threshold was exceeded only in the central part of the Gaussian power distribution. The best results were obtained with hatchings of 0.01 mm and 0.02 mm.

The experimentally determined fluence threshold for laser processing of cellulose fibers was 1.92 × 10^4^ J/m^2^, which corresponds to a pulse energy of 10 µJ. Below this value, no effect was observed. Pulses with energies greater than 34 µJ resulted in strong ablation, which led to rapid evaporation and sublimation of the material. Laser processing results were analyzed for energies in the range of 10–27 µJ.

The effect of laser pulses on cellulose occurs via Fe_2_O_3_ crystals. The laser energy is absorbed by iron, causing a rapid, local temperature increase (up to approximately 2500 K). During beam scanning, a delicate plasma cloud is visible. Thermal energy is transferred to the cellulose in the vicinity of the crystals. This thermal degradation then occurs. This can result in depolymerization, the release of carbon, and the release of CO and CO_2_ gases. These gases escape under high pressure, creating characteristic craters on the fiber’s surface. The optical effect of the laser’s action is the formation of pores on the fiber’s surface and a change in the surface topography.

### 3.3. Roughness Analysis

The table below presents the average values of ten measurements for: R_q_, R_a_, R_max_, R_v_, R_p_ for laser processing with values of 0 W, 3 W, 4 W, and 5 W (Table 7). The values given in the table additionally consider the standard deviation.

Based on the data analysis, it can be concluded that laser power has a direct effect on all tested roughness indices. With increasing power, a systematic and significant increase in the value of each parameter was noticeable, suggesting that increasing laser power causes increasingly deeper and more pronounced changes in the material’s surface structure.

The value of the R_q_ parameter increased from 17 nm (at 0 W) to 70 nm (at 5 W). This increase was almost four times. This means that the surface becomes increasingly diverse in terms of the height of microscopic structures. R_a_ behaved similarly, increasing from 13 nm to 52 nm, which also indicates a deterioration in surface smoothness with increasing energy emitted by the laser beam. The R_a_ parameter is one of the most used indicators in assessing surface quality, and its increase suggests that the laser causes increasingly greater deviations from the ideal plane.

Even more pronounced changes could be observed in parameters describing extreme topographic features, such as R_max_, R_v_, and R_p_. The maximum profile height increased from 127 nm to 576 nm, demonstrating that the difference between the deepest point and the highest peak on the surface was more than three times. This increase demonstrates the increasing amplitude of microstructures because of laser exposure. The maximum pit depth deepened from −60 nm to −306 nm, while R_p_ increased from 68 nm to 270 nm. This indicates that both pits and peaks become increasingly distinct. The surface is intensively thermally and structurally modified. The observed differences result from the physical impact of laser energy on the material. The laser beam introduces thermal energy, which can lead to local melting, expansion, remelting, or even evaporation of the material. At higher powers (4–5 W), this effect is more intense, creating larger deformations, melting zones, and irregularities, and directly affects the surface roughness.

## 4. Conclusions

The laser’s action is absorbed only by the part of the microfiber that consists of magnetically hard material, i.e., iron grain. Cellulose does not absorb laser energy, but the laser causes changes in its structure. The effect of the laser is clearly visible in the appearance of pores on the surface of the fiber. The higher the laser power (in the range from 3 W to 5 W), the larger the gas bubbles. The appearance of pores on the surface may be dangerous as it exposes iron powder, which can crumble and fall out of the fiber, thereby deteriorating the magnetic parameters of such material [43,44].

The appropriate selection of laser power is very important in the surface modification process of electrospun fibers containing iron particles placed in a cellulose matrix. Properly selected radiation energy enables controlled interaction with the material, leading to partial exposure of iron nanoparticles without degrading them or excessively damaging the fiber structure. This phenomenon results from precise energy transfer, which allows for the gentle removal of the cellulose layer surrounding the particles while promoting the reorganization of polymer chains.

The interaction of the laser beam changes the surface structure of the electrospun fibers. Increasing roughness (R_q_, R_a_, R_max_ parameters) in micro- or nanoscale through laser processing can improve the mechanical “anchoring” of the fibers on the substrate or between composite layers. Such irregularities increase the contact surface area, promoting better adhesion, which is particularly important in biomedical applications (implants, tissue scaffolds) or in filtration, where fiber stability is crucial. Increased roughness can also facilitate the binding of functional molecules, nanoparticles, or drugs incorporated into the fiber structure. In pharmaceutical or filtration applications, a larger surface area can improve their effectiveness. Slightly increased surface roughness can promote mechanical stability by dissipating stresses, which can prevent cracking or delamination of the fiber composite. Excessive laser power (4–5 W) causes the roughness parameters to increase very rapidly. This can result in partial melting, deformation, or even rupture of the delicate electrospinning fibers. This can lead to a loss of fiber continuity, which compromises their mechanical and functional properties. Electrospinning allows for the precise formation of fibers with a specific diameter and orientation. Excessive surface roughness and surface damage caused by strong laser exposure can destroy this uniformity, causing undesirable changes in the porosity and structure of the product using electrospinning fibers. High roughness can increase friction or alter the hydrophobicity of the surface, which may not be beneficial in applications requiring smoothness, low absorption, or specific interface properties. Laser processing can be beneficial for electrospinning fibers when used at moderate power (e.g., 3 W in our case or less), allowing for a subtle increase in roughness and improved adhesion or surface properties without damaging the fiber structure. Under these conditions, the functionality of the fiber composite can be improved. However, laser processing at excessively high power can cause excessive surface damage, leading to a significant increase in roughness, and can destroy the continuity and integrity of the fibers, weakening their mechanical and functional properties.

The effect of laser processing depends on the power, wavelength, and exposure time. These parameters must be optimized for each polymer to avoid degradation or undesirable defects. Not all polymers respond to laser processing in the same way. Process optimization is essential.

## Figures and Tables

**Table 1:** Types of modification methods connected with the electrospinning process.

Modification Method	Effect	Citations
Additives in polymer solution	Change fiber shape, add function	[8,9,10]
Adjusting spinning parameters	Control diameter, alignment, porosity	[9,12,13,14]
Post-processing surface functionalization	Add chemical or physical surface properties	[8,12,13]
Post-processing (thermal/solvent)	Modify structure or mechanical properties	[12,13]

**Table 2:** Laser Beam Parameters.

Laser Power [W]	3	4	5
Pulse energy [µJ]	10	13.6	17
Surface power density [W/m^2^]	1.28 × 10^12^	1.71 × 10^12^	2.26 × 10^12^
Fluency [J/m^2^]	1.92 × 10^4^	2.56 × 10^4^	3.2 × 10^4^

**Table 3:** Comparison of SEM images for different values of laser treatment.

Laser Beam Power [W]	Sample No. 1 (10% Ferromagnetic Filling)	Sample No. 2 (23% Ferromagnetic Filling)
0	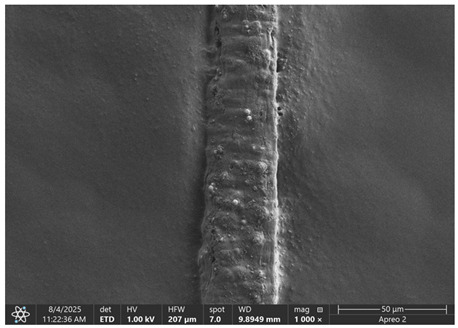	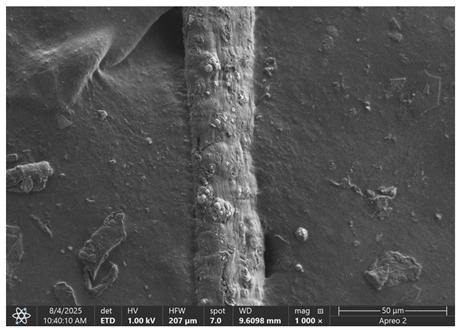
3	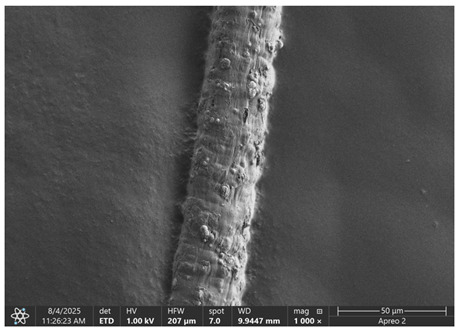	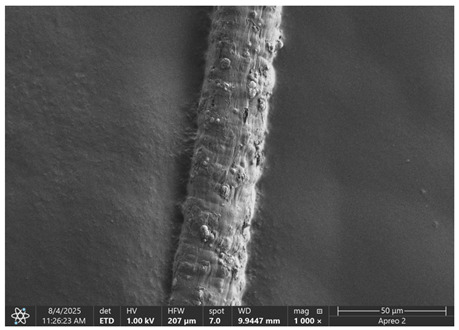
4	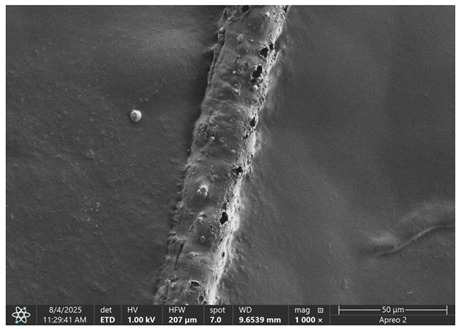	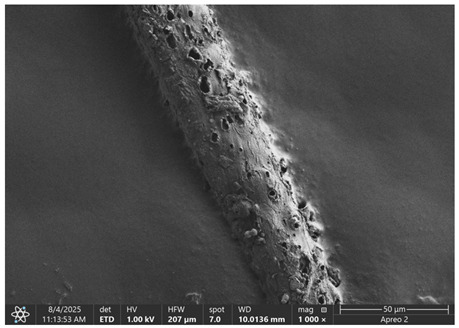
5	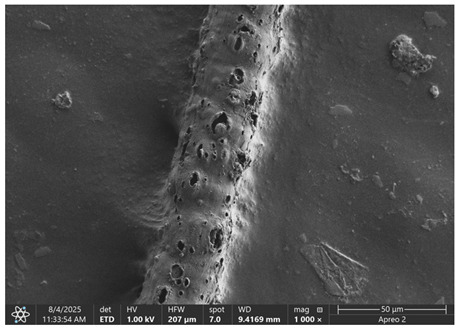	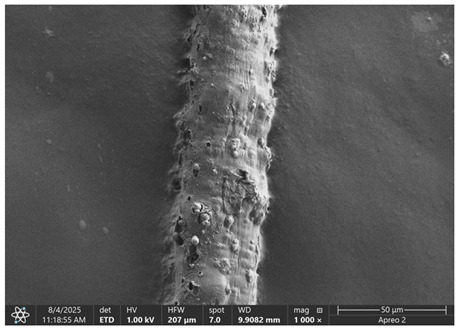

**Table 4:** SEM images of sample No. 1 with a volumetric ferromagnetic content of about 10% for different value of laser treatment.

Laser Beam Power [W]	ETD Detector	T1 Detector
0	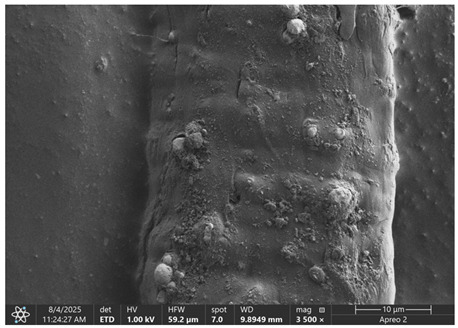	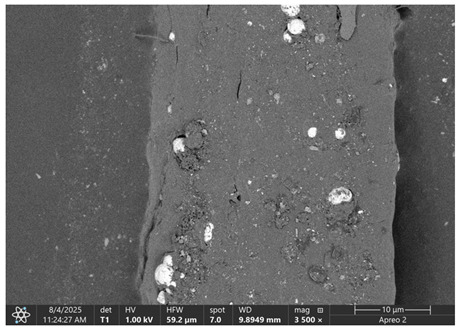
3	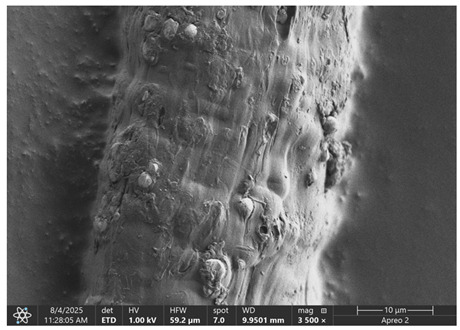	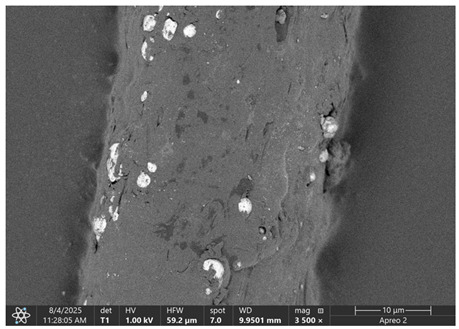
4	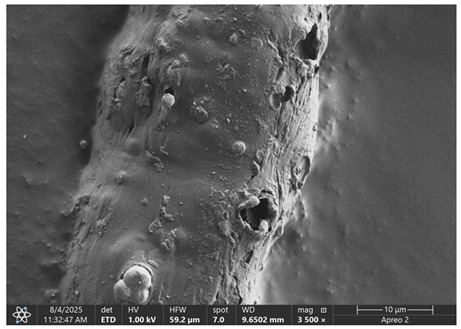	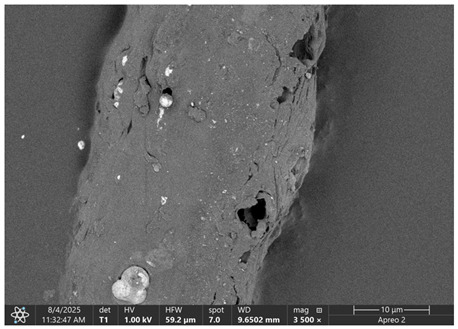
5	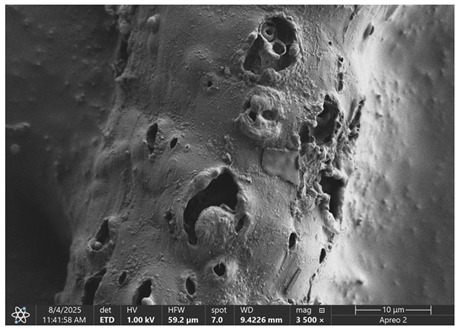	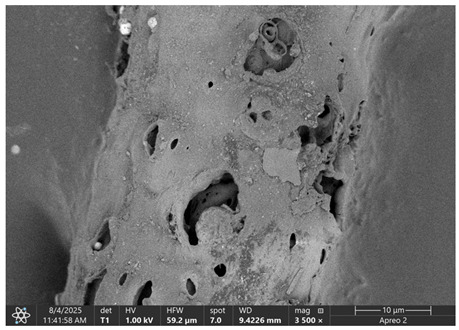

**Table 5:** SEM images of sample no. 2 with a volumetric ferromagnetic content of about 23% for different values of laser treatment.

Laser Beam Power [W]	ETD Detector	T1 Detector
0	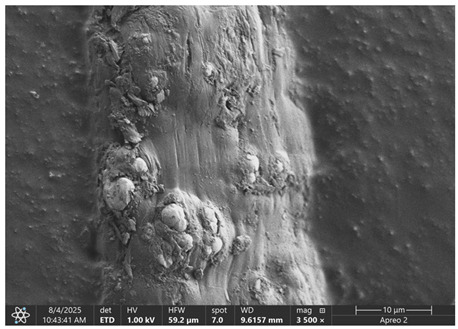	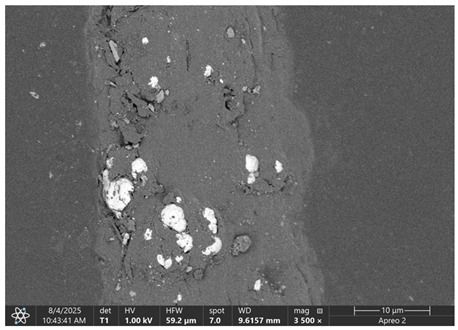
3	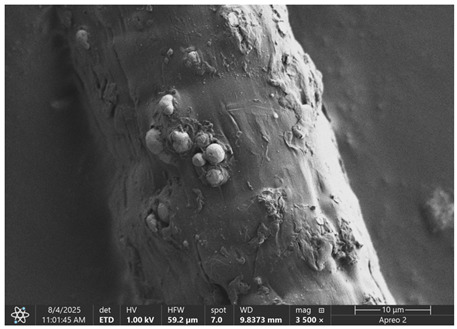	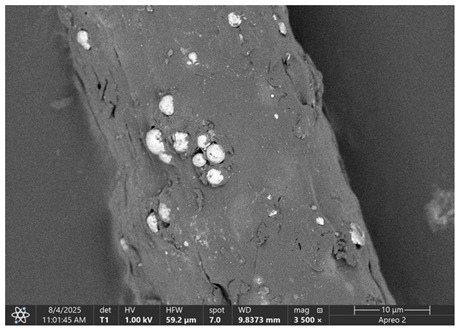
4	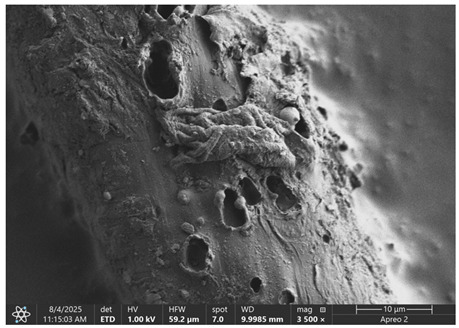	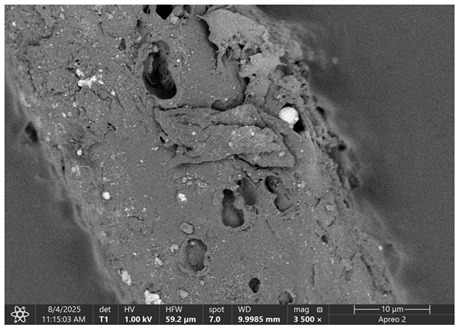
5	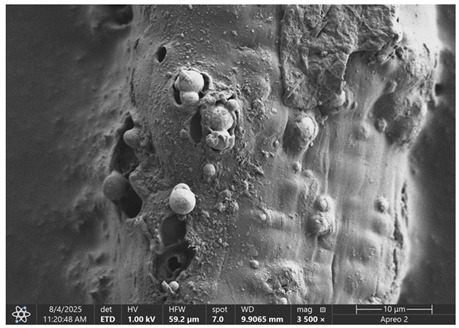	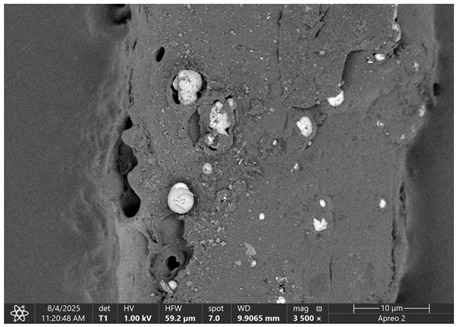

**Table 6:** AFM images of sample no. 1 with a volumetric ferromagnetic content of about 10% for different values of laser treatment. Areas of 3 µm × 3 µm were scanned.

Laser Beam Power [W]	Phase Contrast	Topography (Height)
0	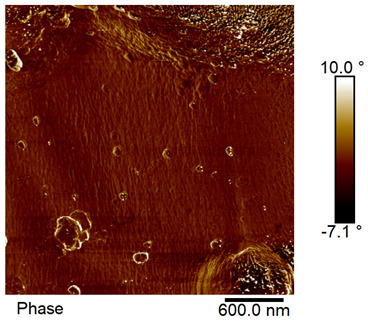	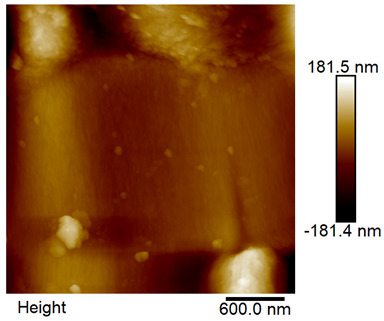
3	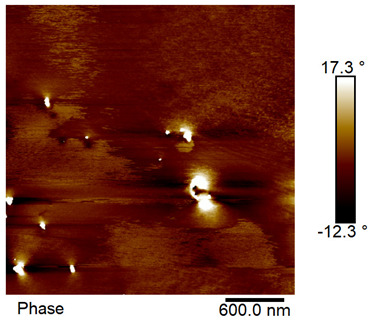	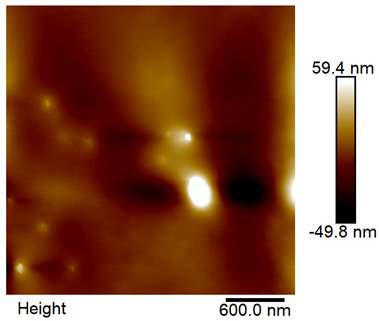
4	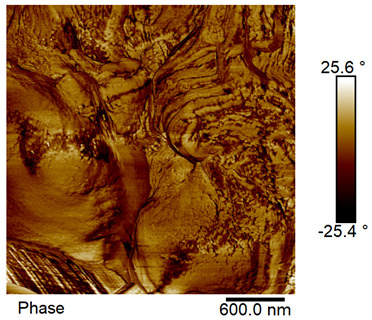	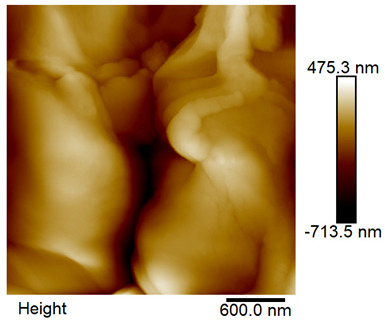
5	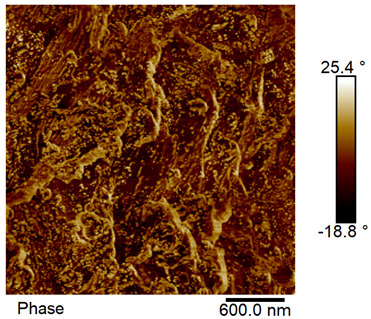	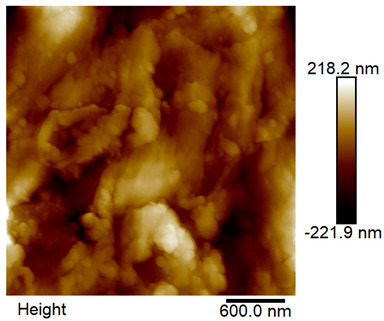

**Table 7:** Roughness values with standard deviation.

Laser Treatment Value [W]	0	3	4	5
R_q_ [nm]	16 ± 6	20 ± 6	20 ± 13	70 ± 37
R_a_ [nm]	13 ± 4	15 ± 6	14 ± 11	52 ± 25
R_max_ [nm]	127 ± 12	194 ± 45	258 ± 132	576 ± 218
R_v_ [nm]	−60 ± 9	−92 ± 60	−123 ± 114	−306 ± 83
R_p_ [nm]	68 ± 3	102 ± 21	134 ± 51	270 ± 142

## Data Availability

The original contributions presented in this study are included in the article. Further inquiries can be directed to the corresponding author.
